# siRNA-Mediated BmAurora B Depletion Impedes the Formation of Holocentric Square Spindles in Silkworm Metaphase BmN4 Cells

**DOI:** 10.3390/insects15010072

**Published:** 2024-01-19

**Authors:** Bing Zhang, Camilo Ayra-Pardo, Xiaoning Liu, Meiting Song, Dandan Li, Yunchao Kan

**Affiliations:** 1 Henan Key Laboratory of Insect Biology in Funiu Mountain, Henan International Joint Laboratory of Insect Biology, College of Life Science and Agricultural Engineering, Nanyang Normal University, 1638 Wolong Road, Nanyang 473061, China; 13261537726@163.com (X.L.); meiting2839@163.com (M.S.); lidannytc@126.com (D.L.); 2 CIIMAR–Interdisciplinary Centre of Marine and Environmental Research, Terminal de Cruzeiros do Porto de Leixões, University of Porto, Avda. General Norton de Matos s/n, 4450-208 Matosinhos, Portugal; cayrapardo73@163.com; 3 School of Life Science and Technology, Henan Institute of Science and Technology, 90 East of Hualan Avenue, Xinxiang 453003, China

**Keywords:** *Bombyx mori*, chromatin-induced spindle assembly, siRNA, cell division, mitosis

## Abstract

**Simple Summary:**

In this study, we explored the assembly of the mitotic spindle in silkworm ovary-derived BmN4 cells, with a specific focus on the role played by a key protein called BmAurora B. Using advanced visualization techniques for cell structures, we examined the architecture and dynamics of spindle microtubules during the early stages of BmN4 cell division. Our investigation showed the formation of a distinctive multipolar square-shaped mitotic spindle, crucial for the accurate segregation of holocentric chromosomes. Intentionally reducing the levels of BmAurora B led to a decrease in the number and size of spindle poles, altering the spindle structure. This change could potentially impact the even distribution of chromosomes between daughter cells. Furthermore, we observed distinct patterns of BmAurora B expression in male and female gonads. This information holds significance as it provides insights into the fundamental processes of cell division in holocentric species. Understanding these mechanisms can contribute to enhancing silk production and advancing silkworm biotechnology. Due to Aurora B’s identification as a cancer cell marker and its association with multipolar spindles in some cancer cells, this research can contribute to broader cancer biology knowledge and may open avenues for advances in cancer diagnostics and therapeutics.

**Abstract:**

Silkworm ovary-derived BmN4 cells rely on chromatin-induced spindle assembly to form microtubule-based square mitotic spindles that ensure accurate segregation of holocentric chromosomes during cell division. The chromosome passenger protein Aurora B regulates chromosomal condensation and segregation, spindle assembly checkpoint activation, and cytokinesis; however, its role in holocentric organisms needs further clarification. This study examined the architecture and dynamics of spindle microtubules during prophase and metaphase in BmN4 cells and those with siRNA-mediated BmAurora B knockdown using immunofluorescence labeling. Anti-α-tubulin and anti-γ-tubulin antibodies revealed faint γ-tubulin signals colocalized with α-tubulin in early prophase during nuclear membrane rupture, which intensified as prophase progressed. At this stage, bright regions of α-tubulin around and on the nuclear membrane surrounding the chromatin suggested the start of microtubules assembling in the microtubule-organizing centers (MTOCs). In metaphase, fewer but larger γ-tubulin foci were detected on both sides of the chromosomes. This resulted in a distinctive multipolar square spindle with holocentric chromosomes aligned at the metaphase plate. siRNA-mediated BmAurora B knockdown significantly reduced the γ-tubulin foci during prophase, impacting microtubule nucleation and spindle structure in metaphase. Spatiotemporal *BmAurora B* expression analysis provided new insights into the regulation of this mitotic kinase in silkworm larval gonads during gametogenesis. Our results suggest that BmAurora B is crucial for the formation of multipolar square spindles in holocentric insects, possibly through the activation of γ-tubulin ring complexes in multiple centrosome-like MTOCs.

## 1. Introduction

In addition to the regular changes in chromatin during the cell cycle, there are also remarkable changes in the non-chromatin phase in the cytoplasm and nucleus. Spindles are dynamic structures composed of microtubule (MT) polymers and hundreds of related factors that can be customized according to the cellular environment. A pair of sister chromatids involved in the regulation of interphase replication is precisely distributed to two daughter cells in the later phase of mitosis [1]. In animal cells containing centrosomes, a pair of right-angled centrioles is near the interphase nuclei. In the S phase, one side of each centriole is joined to form a new centriole [2]. Each pair of centrioles is surrounded by a highly ordered mass of dense material, called pericentriolar material, and together they form the microtubule-organizing centers (MTOCs).

The γ-tubulin ring near the basal foot of each pair of centrioles is the starting point for microtubule assembly [3]. Just before mitosis, short star-shaped radial arrays named asters form around each pair of centrioles. The two centrosomes move apart along the microtubules until they are on opposite sides at the outer edge of the nuclear membrane. When they reach this position, they determine the cell division pole [4]. At this point, the spindles of the two poles are formed. The spindle microtubules that extend from the two poles are called polar microtubules. The microtubules from each pole form half a spindle. Higher plant cells have no centrioles or stars but can still form square-shaped spindles and undergo mitosis due to the presence of substances around the centrioles [5]. In cells without centrosomes, such as female meiotic cells, the chromosomes must nucleate and stabilize microtubules to form a bioriented spindle [6].

Acentrosomal microtubule assembly involves the formation of nuclei near chromosomes or on existing MTs, driven by a RanGTP-dependent mechanism [7]. There are two primary assembly mechanisms: one initiated by chromosomes (i.e., chromosome-mediated spindle assembly) and the other via the augmin pathway, an MT-dependent MT nucleation mechanism [8]. The former mechanism occurs via two pathways that depend on the RanGTP complex [9] and the chromosomal passenger complex (CPC) [10]. The Ran GEF RCC1 protein associates with chromatin and generates a RanGTP concentration gradient that decreases from the center of the chromosome towards the periphery [11,12,13]. By strongly binding importin-β, RanGTP promotes the release of spindle assembly factors (SAFs) from importin-α/-β, facilitating MT nucleation, stability, and assembly around chromosomes. Kinetochore-related CPC, consisting of the mitotic kinase Aurora B in a complex with INCENP, Survivin, and Borealin, promotes MT stability around chromosomes. Aurora B-dependent phosphorylation and inhibition of MT instability factors such as MCAK and OP18 create a favorable microenvironment for MT assembly. Previously, Aurora B CPC was described as playing a crucial role in the maturation of oocytes in *Drosophila melanogaster* and *Xenopus laevis* [14], in MT stabilization, and in spindle formation [15].

BmN4 is a highly heteroploid and polyploid cell line derived from *Bombyx mori* ovarian tissue [16]. The holo-centromere of BmN4 cells has lost the traditional centromeric epigenetic marker CENH3 and the kinetochore protein CENPC [17]. In contrast to the typical monocentric chromosomes, silkworm’s CPC and chromatin-driven spindles are located together on the upper side of the prometaphase chromosomes without forming the typical biaxial spindles. RNAi of the gene encoding Aurora B CPC in BmN4 cells (i.e., BmAurora B) arrests cell cycle progression in prometaphase and disrupts the microtubule network of chromatin-controlled spindles [18]. A recent study compared spindle dynamics and architecture between holocentric BmN4 and monocentric human RPE-1 (retinal pigment epithelial-1) cells during mitosis using live-cell imaging [19]. Notable differences were observed: RPE-1 cells displayed a canonical mitotic phase transition with a bipolar spindle assembled from two foci (centrosomes), while BmN4 cells exhibited distinct features. In BmN4, spindle microtubules sometimes nucleated from more than two foci during prophase, resulting in a square-shaped spindle reminiscent of the square spindle of holocentric plant cells during metaphase. The contribution of BmAurora B to this spindle architecture needs further verification.

In the present study, we examined the architecture and dynamics of spindle microtubules during prophase and metaphase in BmN4 cells and those with siRNA-mediated *BmAurora B* knockdown using immunofluorescence labeling. In addition, we performed qRT-PCR expression analysis of *BmAurora B* in different larval tissues and throughout the fifth larval instar to gain new insights into its regulation during silkworm growth and development. Our results suggest that BmAurora B is crucial for the formation of multipolar square spindles in silkworm holocentric chromosomes, possibly through the activation of γ-tubulin complexes in multiple centrosome-like MTOCs.

## 2. Materials and Methods

### 2.1. Cell Culture and Insects

BmN4 cells from silkworm ovaries were cultured at 27 °C in a TC-100 insect cell culture medium (BBI Life Sciences, Shanghai, China), supplemented with 10% fetal calf serum (Gibco, Invitrogen, Shanghai, China).

The inbred silkworm strain Dazao (P50) was maintained at Nanyang Normal University and reared on fresh white mulberry leaves at 25 ± 2 °C under a 12 h photophase. For spatiotemporal analysis of *BmAurora B* gene expression, more than 30 fifth instar larvae were dissected daily until molting, and samples of the body wall, epidermis, midgut, Malpighian tubules, fat body, silk gland, testis, and ovary were collected, snap-frozen in liquid nitrogen, and stored at −80 °C until use.

### 2.2. Immunofluorescence

BmN4 cells on 12-well glass coverslips were first fixed using 4% paraformaldehyde for 10 min at room temperature, then permeabilized with 0.25% Triton X-100 for 5 min, and subsequently blocked using 3% BSA for 30 min. α-Tubulin staining used α-tubulin mouse monoclonal antibody (Wuhan Sanying Biotechnology Co., Ltd., Wuhan, China) at 1:300 dilution, followed by Alexa Fluor 488-conjugated goat anti-mouse secondary antibody (Jackson ImmunoResearch, Pennsylvania, PA, USA) at 1:1000 dilution. Meanwhile, γ-tubulin was stained with γ-tubulin polyclonal antibody (Wuhan Sanying Biotechnology Co., Ltd.) at 1:400 dilution, followed by Alexa FluorR 594-conjugated goat anti-rabbit secondary antibody (Jackson ImmunoResearch) at 1:1000 dilution. For H3Thr3ph staining, we used a custom rabbit polyclonal anti-H3Thr3ph antibody at 1:500 dilution, obtained by immunizing rabbits with a chemically synthesized peptide AR(pT)KQTARKC of H3Thr3ph protein (GL Biochem, Shangai, China), followed by Alexa FluorR 594-conjugated goat anti-rabbit secondary antibody (Jackson ImmunoResearch) at 1:1000 dilution. In all cases, cells were incubated overnight at 4 °C with the primary antibody and for 2 h at 37 °C with the secondary antibody. Each step was followed by a thorough triple rinse with PBS 1X (137 mM NaCl; 10 mM Na_2_HPO_4_; 1.8 mM KH_2_PO_4_; 2.7 mM KCl; pH 7.4). Finally, nuclei were stained with 4′,6-diamino-2-phenylindole (DAPI; 0.01 mg/mL in 90% glycerol). Fluorescence images were acquired using a ZEISS inverted fluorescence microscope Axio Observer 7 (Carl Zeiss AG, Oberkochen, Germany).

### 2.3. Quantitative RT-PCR (qRT-PCR)

Total RNA was extracted from silkworm samples using TRIZOL (TaKaRa, Dalian, China) and subjected to DNase I treatment to remove potential DNA contamination. Total RNA concentration was measured using the NanoDrop 2000 (Thermo Scientific, Waltham, MA, USA). Complementary DNA (cDNA) was then synthesized from 2 μg of total RNA using the SuperScript™ III First-Strand Synthesis System (Invitrogen, Shanghai, China).

SYBR green real-time PCR master mix (Applied Biosystems, Foster City, CA, USA) was used for 20 μL qRT-PCR reactions. Each reaction contained 10 μL of the 2× concentrated SYBR green real-time PCR master mix, 5 pmol of each primer (Table 1), and 0.2 μg of cDNA template. The PCR was performed in triplicate using an ABI 7500 Real-Time PCR System (Applied Biosystems). The reactions followed a program consisting of incubation at 50 °C for 2 min, then at 95 °C for 15 min, followed by 40 cycles of denaturation at 95 °C for 15 s, annealing at 60 °C for 30 s, and extension and read-out at 72 °C for 30 s. The relative expression levels were calculated using the 2^−ΔΔCt^ method [20]. The silkworm cytoplasmic *actin A3* gene (*Bmactin3*, GenBank accession No. U49854) was used as an internal reference control.

### 2.4. Synthesis of BmAurora B-Specific siRNA Fragments

Four siRNA fragments, sense (-s) and antisense (-as) strands, targeting the open reading frame of *BmAurora B* (GenBank Acc. No. NM_001287844) were designed using the free online tool siDESIGN Center (https://horizondiscovery.com/en/ordering-and-calculation-tools/sidesign-center, accessed on 12 March 2016). The BmAurora B-specific siRNA fragments (siBmAurora B) and a FAM-labeled negative control siRNA (NC-FAM), used for easy monitoring of siRNA uptake by fluorescence microscopy, are shown in Table 1 and were purchased from Oligobio (Beijing, China).

### 2.5. Cell Transfection

The BmN4 cells were inoculated into 12-well cell culture plates 24 h before transfection. The cells were transfected with FAM-labeled NC siRNA and *BmAurora B*-specific siRNA fragments using the transfection reagent LIPO8000 (Beyotime Biotech Company, Shanghai, China), according to the manufacturer’s instructions. A concentration of 50 nM siRNA per well plate was used for transfection. The transfected cells were incubated for 48 h to promote *BmAurora B* turnover. Subsequently, transfection efficiency was assessed using immunofluorescence and qRT-PCR analysis as described above.

### 2.6. Statistics

One-way analysis of variance (ANOVA) in conjunction with Tukey’s post-test at *p* < 0.05 as a significance level was performed to determine differences between siRNA treatments during qRT-PCR analysis. Three biological replicates were used for each experiment, and similar results were obtained. The standard error of the means was used to compare replicates.

## 3. Results

### 3.1. Spindle Microtubule Assembly in BmN4 Cells

**Figure 1:**
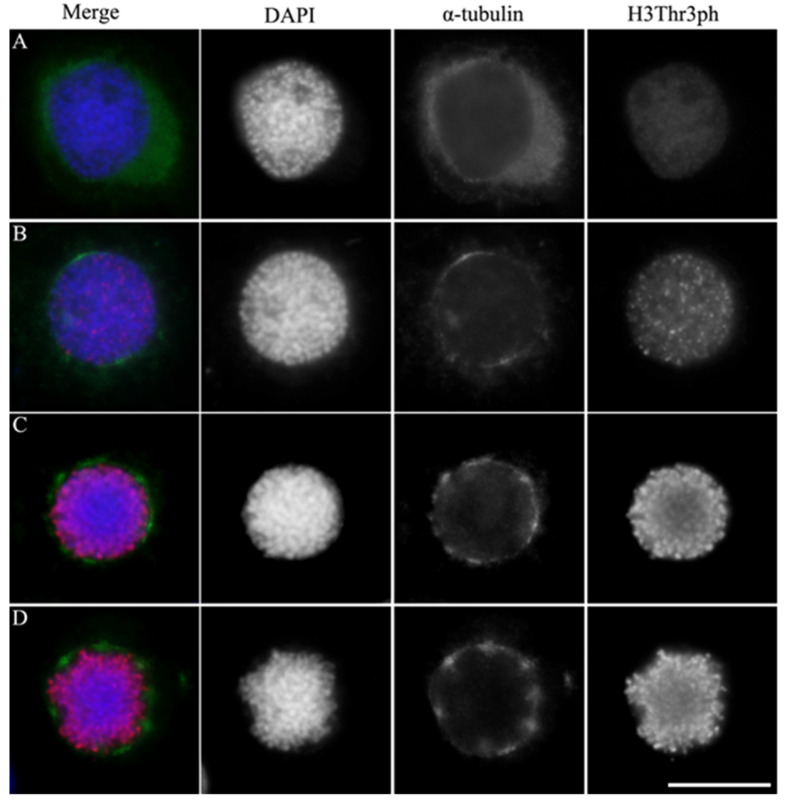
Chromatin-induced spindle assembly in the early prophase of holocentric BmN4 cells. Immunofluorescence staining of α-tubulin (green) and histone H3 Thr3 phosphorylated (H3Thr3ph, red) in prophase (**A**,**B**) and metaphase (**C**,**D**) of mitosis. Panels with the chromatin staining with DAPI in blue were merged (left panels) to show the overlap of the two fluorescence patterns. Scale bar, 5 μm.

In early prophase, immunofluorescence analysis revealed dispersed α-tubulin signals outside the nucleus with an undefined assembly pattern, accompanied by low levels of the chromosome condensation marker, histone H3 Thr3 phosphorylated (H3Thr3ph) (Figure 1A). As prophase progressed, α-tubulin formed a ring-shaped signal with uneven brightness around the nuclear membrane. Simultaneously, scattered H3Thr3ph foci emerged at specific chromatin sites. Notably, the α-tubulin signal was most intense at two particular sites, indicating the onset of spindle microtubule assembly (Figure 1B). In metaphase, α-tubulin had formed bright regions around the nuclear membrane, and H3Thr3ph had distributed across the chromosome (Figure 1C,D). These results corroborate spindle microtubule assembly spanning prophase to metaphase in BmN4 cells.

### 3.2. Colocalization of α-Tubulin and γ-Tubulin Displays Square Spindles in Metaphase BmN4 Cells

**Figure 2:**
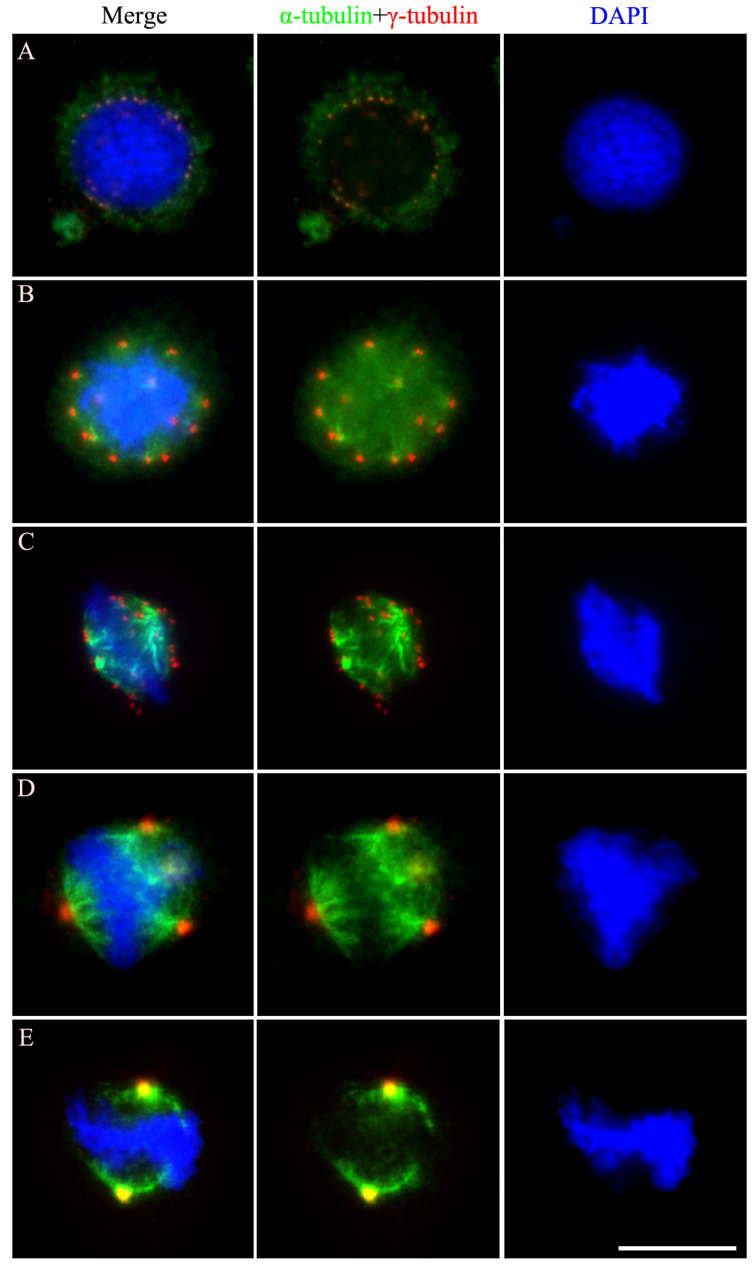
Mitotic spindle assembly dynamics in holocentric BmN4 cells. Immunofluorescence staining of α-tubulin (green) and γ-tubulin (red) in prophase (**A**,**B**) and metaphase (**C**–**E**). Panels with the chromatin staining with DAPI in blue were merged (left panels) to show the overlap of the two fluorescence patterns. Scale bar, 5 μm.

To further investigate spindle assembly in BmN4 cells, we conducted α-tubulin and γ-tubulin colocalization analysis. In early prophase, faint γ-tubulin signals colocalized with α-tubulin were observed during nuclear membrane rupture (Figure 2A). As prophase advanced, γ-tubulin signals increased in both number and intensity. α-Tubulin appeared as multiple bright regions around the nuclear membrane, indicating multiple microtubule-organizing centers (MTOCs). Most MTOCs were observed on the nuclear membrane and surrounding the chromatin, potentially assembling α-tubulin on chromatin (Figure 2B).

In metaphase, the γ-tubulin foci increased in size and number. They scattered on both sides of the chromosomes, forming a multipolar square microtubule spindle with chromosomes aligned to the metaphase plate (Figure 2C). Closer inspection revealed that only part of the α-tubulin accumulated at γ-tubulin sites. As this stage progressed, the square structure became more evident, with chromosomes and spindle microtubules interacting more dynamically. Interestingly, only three to four γ-tubulin foci were detected on both sides of the chromosome of late metaphase cells, which were significantly larger than those previously scattered in early metaphase cells (Figure 2D). The latter suggests possible MTOC fusions, although this needs to be further confirmed. At the end of metaphase, most cells showed a bipolar spindle structure (Figure 2E).

### 3.3. siRNA-Mediated BmAurora B Silencing in BmN4 Cells

**Figure 3:**
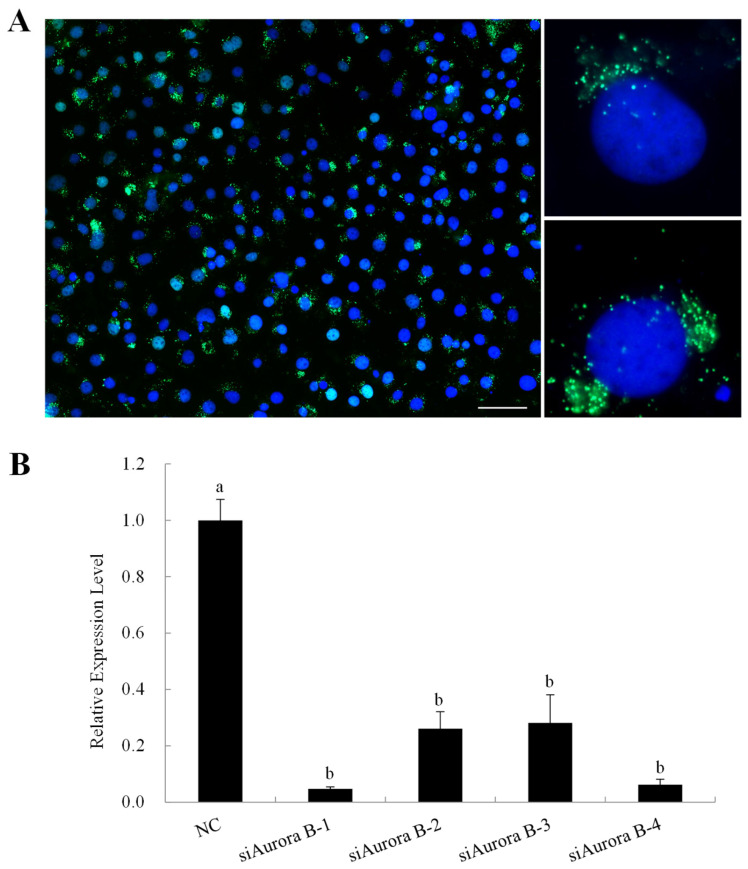
Confirmation of transient transfection efficiency. (**A**) Green fluorescence signals in BmN4 cells transfected with fluorescein amidite (FAM)-labeled negative control siRNA (NC-FAM) for 48 h. Cells were stained with DAPI in blue. Scale bar, 5 μm. (**B**) Real-time quantitative polymerase chain reaction (qRT-PCR) of BmAurora B after the transfection of specific siRNA (siAurora B-1, siAurora B-2, siAurora B-3, and siAurora B-4) and NC-FAM (NC). The silkworm *actinA3* gene was used as an internal control. Bars represent means ± SE. The means were compared using a one-way ANOVA test with Tukey’s post-test at *p* < 0.05. Treatments not sharing a common letter were significantly different (*p* < 0.05).

BmN4 cells were transfected with fluorescein amidite (FAM)-labeled negative control siRNA (NC-FAM) and four distinct siRNA fragments targeting various regions of BmAurora B mRNA. Forty-eight hours post-transfection, numerous dot-shaped green fluorescent signals from NC-FAM were evident in the cytoplasmic region outside the nuclear membrane (Figure 3A), indicating successful siRNA fragment introduction.

The qRT–PCR results showed a significant decrease in *BmAurora B* mRNA levels with each specific siRNA fragment. While there were no significant differences in knockdown levels between the four siBmAurora B fragments, both siAurora B-1 and siAurora B-4 showed the greatest decrease in *BmAurora B* transcripts, achieving an approximately 85% reduction (Figure 3B). Consequently, these two fragments were selected for further functional analysis.

### 3.4. BmAurora B-Depleted Cells Do Not form Square Spindles in Metaphase

**Figure 4:**
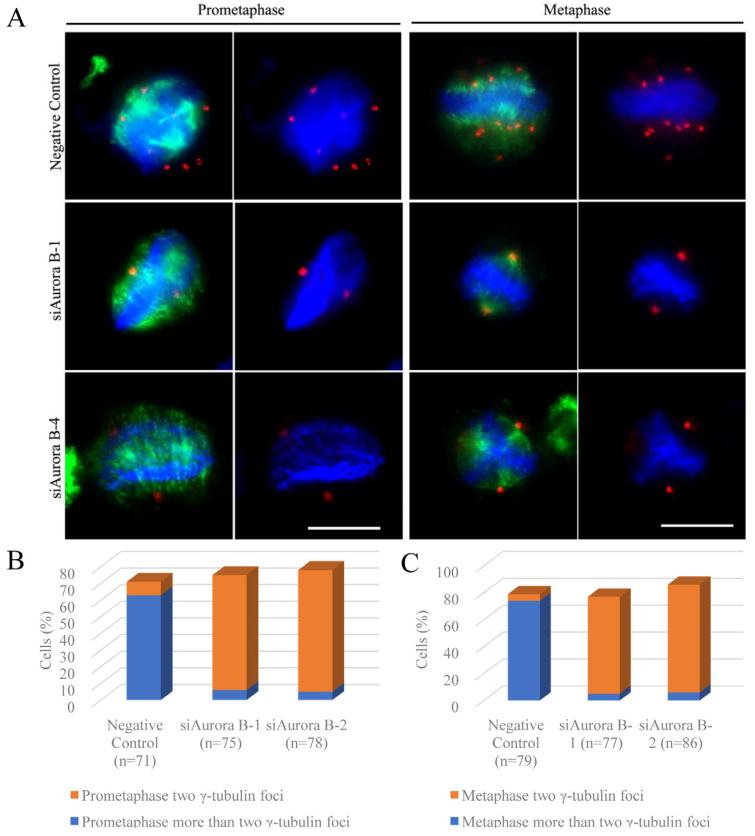
Effects of siRNA-mediated BmAurora silencing B on the mitotic spindle assembly dynamics in holocentric BmN4 cells. (**A**) Immunofluorescence staining of alpha-tubulin (green) and gamma-tubulin (red) signals in siRNA-transfected BmN4 cells in prometaphase and metaphase. Chromatin was stained with DAPI in blue. Scale bar, 5 μm. (**B**) Percentage of prometaphase cells showing different γ-tubulin foci (*n* = number of cells analyzed per condition). (**C**) Percentage of metaphase cells showing different γ-tubulin foci (*n* = number of cells analyzed per condition).

We investigated spindle formation in siAurora B-1- and siAurora B-4-transfected BmN4 cells during prophase and metaphase through the colocalization of α-tubulin and γ-tubulin. In prophase, depletion of BmAurora B significantly reduced γ-tubulin complexes, which are critical for spindle microtubule assembly near chromatin. Furthermore, α-tubulins were scattered in siRNA-mediated BmAurora B-depleted cells with obvious difficulties in microtubule nucleation compared to NC-FAM-transfected cells (Figure 4A, left panels).

In metaphase, more than 92% of siAurora B-transfected cells showed only two γ-tubulin foci (Figure 4B,C), indicative of spindle poles, in contrast to control cells with multiple γ-tubulin foci distributed on both sides of the chromosome forming a multipolar, square microtubule spindle with chromosomes aligned to the metaphase plate. In BmAurora B-depleted cells, microtubules faced challenges in nucleation, resulting in chromosomes poorly aligned to the metaphase plate. As a consequence, the resultant spindle structure took on a quasi-form, closely resembling the canonical oval spindle typically observed in monocentric organisms (see Figure 4A, right panels).

### 3.5. Spatiotemporal Analysis of BmAurora B Expression in Silkworm Larval Tissues

**Figure 5:**
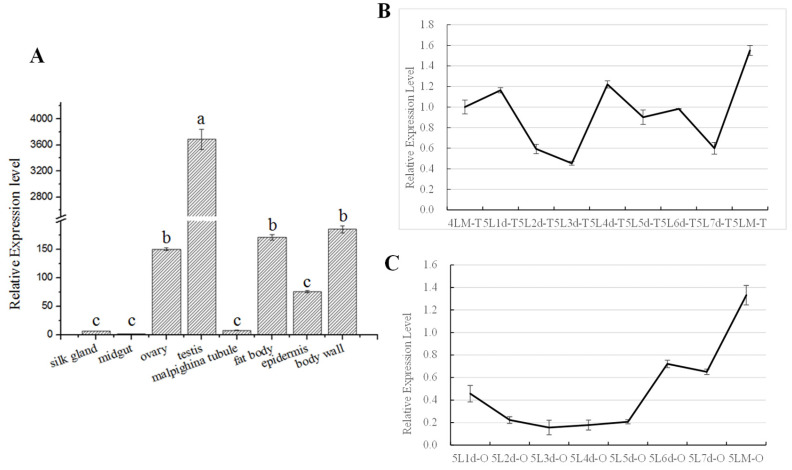
Spatiotemporal *BmAurora B* expression analysis in 5th instar larvae of *Bombyx mori* by qRT-PCR. (**A**) Quantification of *BmAurora B* mRNA in multiple tissues of silkworm larvae on day 3 of the fifth instar (5L3d). Bars represent means ± SE. The means were compared using a one-way ANOVA test with Tukey’s post-test at *p* < 0.05. Treatments not sharing a common letter were significantly different (*p* < 0.05). (**B**) Quantification of *BmAurora B* mRNA in the testis from day 1 of the 5th instar larvae (5L1d) to the molting stage (5LM). (**C**) Quantification of *BmAurora B* mRNA in the ovary from day 1 of the 5th instar larvae (5L1d) to the molting stage (5LM). The values represent the relative mRNA expression with respect to the internal control. The data represent the mean ± SE of the triplicates.

To gain new insights into the role of *BmAurora B* in insect growth and development, we analyzed its expression pattern in different tissues of silkworm larvae at the fifth (last) instar stage using quantitative RT-PCR. The results showed that *BmAurora B* was highly expressed in the testis, followed by the body wall, ovary, and fat body, while the expression was very low in the silk gland, midgut, Malpighian tubules, and epidermis of the larvae on the third day of the fifth instar (5L3d) (Figure 5A). Further analysis of *BmAurora B* expression in the testis and ovary during the entire fifth instar stage showed fluctuating accumulation in the testis (Figure 5B) and progressive accumulation in the ovary (Figure 5C), with both reaching the highest levels at the onset of ecdysis.

## 4. Discussion

In the present study, we have characterized the multipolar square spindle of mitotic BmN4 cells using immunofluorescence experiments. The multipole formed by γ-tubulin ring complexes eventually fused into a bipolar or near-bipolar state, which is crucial for the successful division of later sister chromatids into two poles. Previously, Mon et al. (2014) [18] observed that the spindle in BmN4 cells of silkworm was positioned on the upper side of the prometaphase chromosome, and α-tubulin antibody immunofluorescence displayed typical biorientation at metaphase. These findings align with the positioning results of α-tubulin obtained in our study. However, our α-tubulin and γ-tubulin colocalization analysis revealed that chromatin-mediated spindle assembly in metaphase BmN4 cells was multipoint, with multiple γ-tubulin foci still present.

The formation of multipolar square spindles in mitotic BmN4 cells was impaired after siRNA-mediated knockdown of the *BmAurora B* gene. In a *Drosophila* S2 cell line, silencing of the *Aurora B* gene resulted in multipolar spindle formation in some cells in early mitosis and reduced microtubule density in the central region of the spindle in late mitosis, preventing cytokinesis and polyploid formation [21]. This shows that the *Aurora B* gene is involved in spindle microtubule assembly. The different phenotypes in the BmN4 and S2 cell lines could be associated with the latter having monocentric chromosomes, whereas the chromosome of BmN4 cells is a diffuse centromeric chromosome without CENH3 or CENPC. In *Caenorhabditis elegans*, a nematode with the same diffuse centromeric chromosome, Aurora B, even when inactivated after mitosis, disrupts cytokinesis and causes defects in the apical structure, suggesting that it is involved in the regulation of epithelial polarization after cytokinesis [22]. Aurora B can also promote sister chromatid adhesion release in late-stage hypothyroid oocytes by phosphorylating the adhesion protein subunit Rec8 [23]. Recent studies have shown that the kinase activity of Aurora B is crucial for acentrosomal-mediated bipolar spindle assembly in nematode cells [24]. In our study, siRNA-treated cells showed a decrease in the number of γ-tubulin foci and high α-tubulin dispersion, indicating obvious difficulties in microtubule nucleation compared to untreated control cells. These results suggest that BmAurora B-mediated phosphoregulation is crucial for the organization of multipolar square spindles in holocentric BmN4 cells, not described previously, probably through direct or indirect phosphorylation of downstream effectors involved in the de novo assembly of γ-tubulin ring complexes and spindle formation.

In BmN4 cells, mitotic microtubule generation depends on the formation of the γ-tubulin ring complex. This protein complex, composed of γ-tubulin complex proteins (GCP family proteins), forms connections between its components, creating a structure in which a ring of γ-tubulin molecules mimics the plus end of a microtubule, serving as a crucial starting point for microtubule assembly [25]. Mitotic kinases are involved in the activation of several GCP proteins that regulate various processes at the γ-tubulin ring complexes, such as biogenesis and the duplication of centrioles [26,27].

Some spindle proteins, particularly the motor kinesin proteins, are also regulated by mitotic kinases. In *Caenorhabditis elegans*, phosphorylation of Kin-5 by Aurora B kinase is crucial for its activation [28]. Interestingly, Kin-5 and Kin-14 have already been shown to be important orchestrators that play a crucial role in the organization of the square spindle assembly in BmN4 cells [29]. In addition, Aurora B can phosphorylate and activate other kinases during mitosis, such as the CPC INCENP [30] and Polo-like kinase 1 (PlK1), which in turn phosphorylates mitotic centromere-associated kinesin (MCAK) to stimulate its microtubule depolymerase activity [31]. In both cases, the cascade of phosphorylation processes is crucial for accurate chromosome segregation. In another example, the microtubule-destabilizing Oncoprotein 18/Stathmin (Op18) protein is phosphorylated by Aurora B during spindle assembly [32]. Surprisingly, an increase in Aurora B activity is also detrimental to cells, and, in yeast, it causes defects in chromosome segregation and spindle assembly checkpoint activation [33]. This interconnected network of molecular interactions clearly demonstrates the complexity and precision underlying the regulation of microtubule assembly, in which phosphorylation processes involving the mitotic kinase BmAurora B are of fundamental importance.

Aurora B is constitutively expressed in mitotically active cells and up-regulated in highly proliferative tissues. A previous study [34] revealed high expression of the *BmAurora B* gene in the testis and ovary of silkworms during the final larval instar. This coincided with the cell proliferation phase of both female and male gametogenesis in silkworms. Building on this research, in our study, we investigated the kinetics of *BmAurora B* expression during the last larval stage in more detail. In particular, we found different *BmAurora B* mRNA accumulation patterns in the testis and ovary, indicating differences in mitotic progression between these tissues.

Aurora B kinases, displaying remarkable sequence similarity throughout evolution, possess a highly conserved catalytic domain. This domain exhibits serine/threonine kinase activity, acquired through auto-phosphorylation of a conserved residue at Thr232 upon binding of a co-factor during mitosis [35]. This mitotic kinase plays a central role in orchestrating mitotic progression and exerts its influence by phosphorylating multiple substrates. Mass spectrometry analyses of HeLa cells arrested in the G1 and mitotic phases of the cell cycle have unveiled a staggering array of over 1000 distinct mitotic-regulated phosphoproteins, shedding light on the extensive regulatory network governed by Aurora B in these cells [36]. While similarities and differences in the function of Aurora B between monocentric and holocentric organisms have been described [37], unknown facets remain to be explored. In this context, future studies on the phosphoproteins in BmN4 cells could help to identify new BmAurora B targets to unravel the intricacies of the functions of this kinase during mitosis in holocentric species, providing valuable insights into the broader landscape of cell division regulation.

In summary, we have shown that BmAurora B is crucial for the formation of multipolar square spindles in holocentric BmN4 cells, possibly through the activation of γ-tubulin complexes, and we found that its high expression in the gonads of the last instar larvae follows distinct patterns. Given Aurora B’s identification as a marker for cancer cells and the presence of multipolar spindles in some cancer cells, further exploration of BmAurora B’s functions in BmN4 mitosis may offer promising avenues for advances in cancer diagnostics and therapeutics.

## Figures and Tables

**Table 1:** Primers used in this study.

Primer	Primer Sequence (5′-3′)	Purpose
BmAurora B-RT-F	GGCCAAGGCAAATTCGGACATGTT	qRT-PCR
BmAurora B-RT-R	CGTCCTTGGGGTGAATTTGTGAGATGTT	
BmA3-RT-F	ATGTGCGACGAAGAAGTTGC	
BmA3-RT-R	GTCTCCTACGTACGAGTCCT	
siBmAurora B-1-s	CCAGAAAGUAAAGCAGCAATT	siRNA synthesis
siBmAurora B-1-as	UUGCUGCUUUACUUUCUGGTT	
siBmAurora B-2-s	GGGAAAGCCUCCAUUUGAATT	
siBmAurora B-2-as	UUCAAAUGGAGGCUUUCCCTT	
siBmAurora B-3-s	CCUGAUGGAGCCAAGGAUUTT	
siBmAurora B-3-as	AAUCCUUGGCUCCAUCAGGTT	
siBmAurora B-4-s	GCCAAGGAUUUGAUCUCAATT	
siBmAurora B-4-as	UUGAGAUCAAAUCCUUGGCTT	
NC-FAM-s	UUCUCCGAACGUGUCACGUTT	
NC-FAM-as	ACGUGACACGUUCGGAGAATT	

## Data Availability

The data presented in this study are available in the article.
